# Creatine kinase rate constant in the human heart at 7T with 1D-ISIS/2D CSI localization

**DOI:** 10.1371/journal.pone.0229933

**Published:** 2020-03-19

**Authors:** Adil Bashir, Jianyi Zhang, Thomas S. Denney

**Affiliations:** 1 Department of Electrical and Computer Engineering, Auburn University, Auburn, Alabama, United States of America; 2 Department of Biomedical Engineering, The University of Alabama at Birmingham, Birmingham, Alabama, United States of America; McLean Hospital, UNITED STATES

## Abstract

**Purpose:**

Creatine Kinase (CK) reaction plays an important role in energy metabolism and estimate of its reaction rate constant in heart provides important insight into cardiac energetics. Fast saturation transfer method ($T_{1}^{nom}–T1$ nominal) to measure CK reaction rate constant (*k*_*f*_) was previously demonstrated in open chest swine hearts. The goal of this work is to further develop this method for measuring the *k*_*f*_ in human myocardium at 7T. $T_{1}^{nom}$ approach is combined with 1D-ISIS/2D-CSI for in vivo spatial localization and myocardial CK forward rate constant was then measured in 7 volunteers at 7T.

**Methods:**

$T_{1}^{nom}$ method uses two partially relaxed saturation transfer (ST) spectra and correction factor to determine CK rate constant. Correction factor is determined by numerical simulation of Bloch McConnell equations using known spin and experimental parameters. Optimal parameters and error estimate in calculation of CK reaction rate constant were determined by simulations. The technique was validated in calf muscles by direct comparison with saturation transfer measurements. $T_{1}^{nom}$ pulse sequence was incorporated with 1D-image selected in vivo spectroscopy, combined with 2D-chemical shift spectroscopic imaging (1D-ISIS/2D-CSI) for studies in heart. The myocardial CK reaction rate constant was then measured in 7 volunteers.

**Results:**

Skeletal muscle *k*_*f*_ determined by conventional approach and $T_{1}^{nom}$ approach were the same 0.31 ± 0.02 s^-1^ and 0.30 ± 0.04 s^-1^ demonstrating the validity of the technique. Results are reported as mean ± SD. Myocardial CK reaction rate constant was 0.29 ± 0.05 s^-1^, consistent with previously reported studies.

**Conclusion:**

$T_{1}^{nom}$ method enables acquisition of ^31^P saturation transfer MRS under partially relaxed conditions and enables 2D-CSI of *k*_*f*_ in myocardium. This work enables applications for in vivo CSI imaging of energetics in heart and other organs in clinically relevant acquisition time.

## Introduction

Energy is required for normal cardiac function and alterations in energy metabolism may contribute to the development of heart failure [1–4]. Phosphorus (^31^P) magnetic resonance spectroscopy (MRS) provides the only non-invasive means to monitor high energy phosphate metabolites in the myocardium. Creatine phosphate (PCr) and adenosine triphosphate (ATP) are two prominent signals in cardiac ^31^P MRS. A decrease in PCr/ATP ratio has been used as an early indicator of energy deficiency and may predict mortality in heart failure patients. However, the PCr/ATP ratio cannot detect decrease in ATP concentration which are only observed in advanced heart failure [5–7].

The amount of ATP stored in the heart is small and there is a constant need for ATP production and transport. The amount of ATP produced and consumed is many times greater than the ATP pool stored in the heart hence an estimate of ATP turnover may provide a better estimate of energy supply in the heart. The creatine kinase system plays an important role in this regard and acts as an energy reserve and transport mechanism, ensuring that there is an abundant and immediate supply of ATP for heart function. The CK system reversibly converts adenosine diphosphate (ADP) and phosphocreatine (PCr) to ATP and creatine (Cr):

$$PCr+ADP+H^{+}⟺Cr+ATP$$ (1)

Alterations in the CK system are seen early in heart failure, suggesting impaired delivery of ATP to energy-consuming systems. This implies that flux through the CK reaction may be a more sensitive marker of myocardial energy deficiency compared with the PCr/ATP ratio [5, 8–12]. ^31^P saturation transfer MRS allows measurement of the forward creatine kinase reaction rate constant (PCr → ATP). Combined with absolute quantification of PCr concentration, the flux of ATP production through the CK reaction can be calculated [13, 14].

^31^P MRS suffers from low signal to noise and the conventional ^31^P-MRS saturation transfer method requires lengthy data acquisition time. This prohibits the spatially localized measurements of CK reaction rate constants in vivo. Several techniques have been developed to optimize the acquisition strategy for speedier CK flux measurements [15–21]. These techniques rely on pulse sequences that provide little spatial localization [21] or 1D-CSI spatial localization in the myocardium [15–17]. Triple repetition time saturation transfer (TriST) and Two repetition time Saturation Transfer (TwiST) require acquisition with long TR and have been successfully demonstrated with 1D-CSI localization in myocardium. 2D and 2D CSI implementations or these approaches would make the scan prohibitively long for human studies. Bloch-Siegert four Angle Saturation Transfer (BOAST), a variant of Four Angle Saturation Transfer (FAST), has been recently implemented at 7T to measure 3D spatially localized CK reaction rate constant in human myocardium [20]. The approach acquires ^31^P spectra at known flip angles to calculate the CK reaction rate constant. Since surface coils are primarily used in cardiac spectroscopy the flip angles across myocardium can vary significantly. To overcome this limitation the technique relies on Bloch-Siegert based flip angle mapping technique using off resonance Fermi pulses to determine the flip angles [22, 23]. The low SNR of ^31^P, and phase inhomogeneity in cardiac studies at 7T makes accurate determination of flip angles technically challenging. In addition, the 3D-CSI localization can result in long acquisition times and a poor point spread function. This work is aimed to present a simple approach to measure localized in vivo CK reaction rate constants in the human myocardium at 7T.

We have previously established a steady-state saturation transfer method ($T_{1}^{nom}$) for performing rapid measurements of CK in open chest swine hearts [18]. Here we sought to translate the methodology to for the measurement of spatially localized CK reaction rate constant in human hearts at 7T. The novelty of this approach is the 2D mapping of CK flux in human heart in clinically relevant exam time. It also addresses technical challenge for in vivo localization of ^31^P MRS with surface coil. It is the first application to demonstrate 2D spatial localization in heart using broadband Gradient Modulated Independent Adiabaticity with WURST modulation (GOIA-WURST) allowing excellent slice selection with a surface coil [24–26]. The protocol is developed with extensive numerical simulations to provide error analysis of various physiological and experimental factors affecting the reaction rate measurements.

The $T_{1}^{nom}$ strategy was validated in skeletal muscle, which generated forward pseudo-first-order CK reaction rate constant (*k*_*f*_) values that were not statistically different from values measured with the conventional approach. We then used $T_{1}^{nom}$ to measure the CK rate constant in human myocardium at 7T. This technique offers the opportunity for longitudinal studies, allowing repeated measurements within the same animal or subject.

## Theory

The creatine kinase enzyme catalyzes the exchange of ^31^P spins between PCr and γ-ATP. The evolution of magnetization using a two-site (Eq 1) chemical exchange can be modeled by the Bloch-McConnell equations [27]. In saturation transfer experiments, where γ-ATP is selectively saturated, the equation describing the evolution of PCr magnetization is given by

$$\frac{dM_{PCr}(t)}{dt}=\frac{M_{o}-M_{PCr}(t)}{T_{1}^{int}}-k_{f}M_{PCr}(t)$$ (2)

here *M*_*o*_ represent the fully relaxed PCr magnetization without saturation of γ-ATP, $T_{1}^{int}$ is the intrinsic longitudinal relaxation time constants of PCr in absence of exchange, and *k*_*f*_ is the pseudo first order reaction rate constant of the CK shuttle. This equation describes the unidirectional kinetics of ATP production from PCr. From the solution to the above equation, it can be shown that

$$\frac{M_{o}}{M_{ss}}=k_{f}T_{1}^{int}+1$$ (3)

where *M*_*ss*_ is the steady state fully relaxed magnetization with saturation of γ-ATP. This equation is similar to that as used in TriST approach [16]. TriST requires a fully relaxed (long repetition time, TR) scan and would have prohibitively long duration experiments for 2D CSI [22]. Numerous studies have shown that $T_{1}^{int}$ is similar among patients, healthy individuals and even among different species [28–32]. In order to improve efficiency and signal to noise (SNR) per unit time the spectra need to be acquired with short repetition time—i.e. partially relaxed spectra. Under these partially relaxed conditions, the ratio of PCr magnetization without (*M*_*c*_) and with (*M*_*s*_) γ-ATP saturation still has an approximately linear relationship with *k*_*f*_, and is given by [18]

$$\frac{M_{c}}{M_{s}}\approx k_{f}T_{1}^{nom}+1$$ (4)

**Fig 1:**
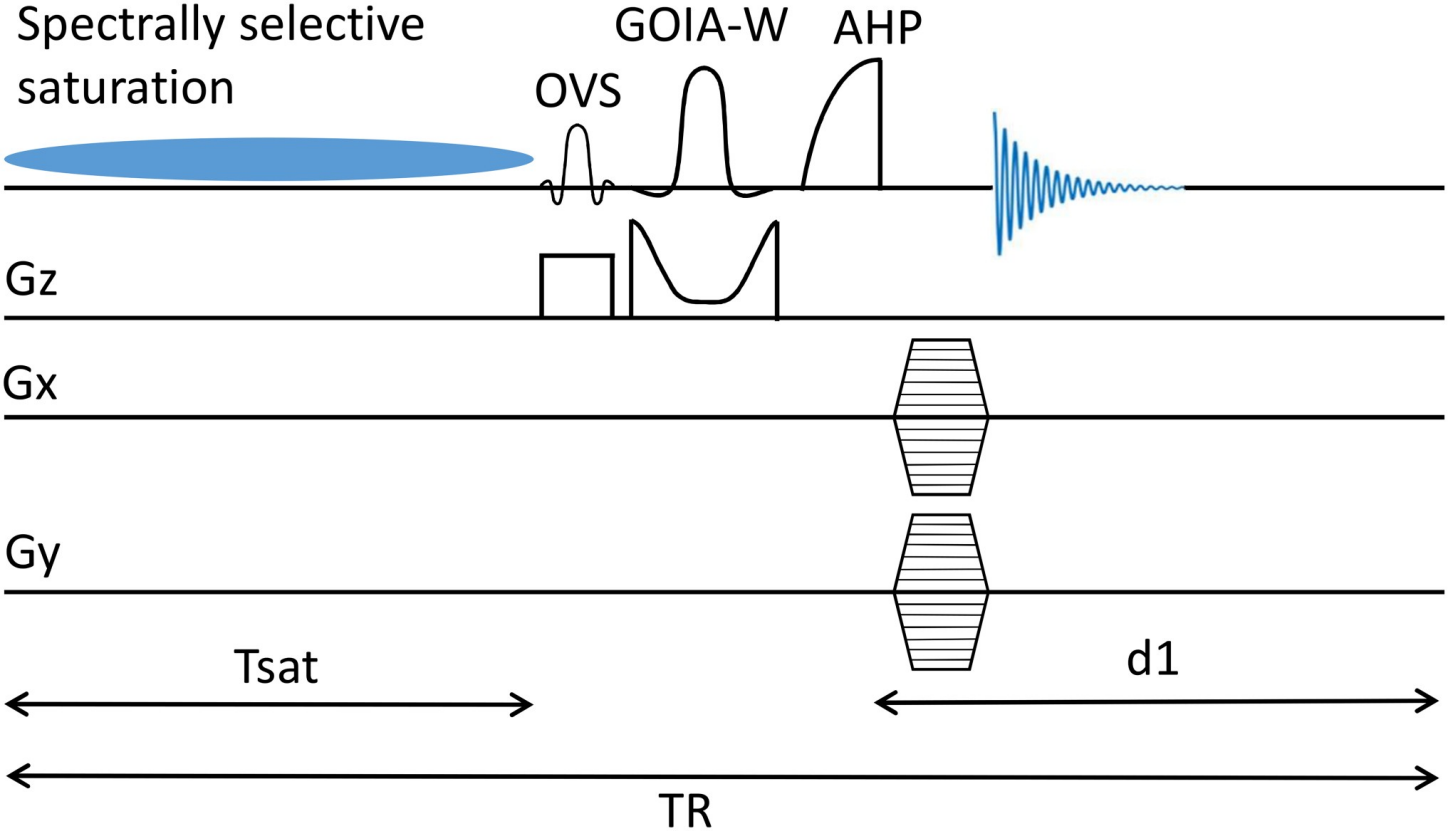
Pulse sequence for saturation transfer study. *T*_*sat*_ is the duration of saturation pulse and d1 is the delay for recovery of magnetization. The GOIA-WURST (16, 4) inversion pulse is applied in alternate scans for each phase encoding step. Time axis is not to scale.

Where the $T_{1}^{nom}$ (*T*_*1*_ nominal) parameter is a function of spin parameters (relative pool size of metabolites and their respective $T_{1}^{int}$) and experimental acquisition parameters (TR, T_sat_, and flip angle) [18]. There is no general analytic equation which describes $T_{1}^{nom}$ however, it can be determined by simulation of Bloch McConnell equation (Eq 1) given the pulse sequence acquisition parameters and system spin parameters. A schematic of the pulse sequence used is shown in Fig 1. *M*_*c*_ and *M*_*s*_ were numerically determined for flip angle of 90°, TR = 2.8 s and T_sat_ = 1.8 s and spin parameters were selected based on data at 7T: *PCr*/*ATP* = 2, $T_{1,PCr}^{int}=4.5s$, $T_{1,ATP}^{int}=1.8\;s$ and *k*_*f*_ was allowed to vary from 0 to 5.5 s^-1^ [19].

## Methods

All measurements were performed on 7T Magnetom system (Siemens, Erlangen, Germany) using a double tuned ^1^H/^31^P surface coil with 11 cm diameter ^31^P inner loop and 12.5 cm diameter ^1^H outer loop (RAPID Biomedical GmbH, Germany). Studies were approved by the Institutional Review Board at Auburn University, Auburn, AL USA and participants provided written informed consent.

The schematic diagram of the pulse sequence is shown in Fig 1. The sequence consists of a fully adiabatic ^31^P 2D-CSI sequence with interleaved 1D-ISIS/2D-CSI selection. Slice localization scheme consisted of two acquisitions. For the first acquisition the spins in the selected slice are inverted with 5 ms inversion pulse (GOIA-WURST (16, 4)) with 6 kHz bandwidth [25, 33, 34]. For the second acquisition the slice selection pulse was turned off. Slice localization is achieved by subtracting the two acquisitions. A GOIA pulse was used because it can provide a good excitation uniformity and has a very wide bandwidth, which reduces chemical shift displacement error (CSDE). GOIA-WURST pulses with bandwidth 6 kHz leads to CSDE of 5% in the slice direction between PCr and γATP [35, 36]. To acquire a complete 2D CSI dataset, the GOIA pulse was turned on/off for each phase encoding step before proceeding to the next phase encoding step. A frequency selective saturation pulse preceded the slice selection and phase encoding. The frequency selective saturation pulse consisted of a 99 ms duration low power hard pulse with saturation voltage was set at ~18 Hz. The saturation times are achieved by looping over the saturation pulse with a 1ms gap in between. A 2.56 ms AHP pulse was used for excitation [37]. Before proceeding with the in vivo experiments, the slice section profile of the implemented GOIA-WURST (16, 4) inversion pulse was determined experimentally in a cylindrical sodium phosphate phantom (S1 Fig). The suppression of contaminating signals arising from chest muscles was achieved by spatially selective outer volume suppression (OVS) RF pulse inserted into the main pulse sequence between spectral saturation pulses and slice selection pulses. Optimization of the OVS pulse was done in a phantom and used for in vivo studies without further calibration (S2 Fig). The effectiveness of OVS in vivo was tested by measuring global spectra with and without OVS (S3 Fig).

Phantom experiments were performed to validate the performance of the RF pulse as a function of depth. A vial (1.8 cm x 2.4 cm x 3.6 cm) containing sodium phosphate was placed inside a large beaker full of sodium chloride. Saturation recovery pulse sequence (TR = 2, 4, 8, 12, 20, 30 s) was used to measure the T1 in the vial at a distance of 2.5 cm and 6 cm from the center of the RF coil and compared with 12 point inversion recovery method with TR = 50 sec. These data was acquired on resonance and at -150 Hz, and +150 Hz off resonance.

### Skeletal muscle validation

Validation of $T_{1}^{nom}$ method was performed in calf muscles of 3 subjects. The $T_{1}^{nom}$ method was implemented with GOIA-1D-ISIS/1D-CSI with the CSI grid in plane perpendicular to the surface of the coil placed under the calf. A saturation pulse with duration *T*_*sat*_ = 1.8s was used to saturate γ-ATP resonance and a recovery time *d*_*1*_ = 1s. The other acquisition parameters were: spectral width = 6 kHz, FOV = 16 cm, phase encoding steps = 16, averages = 16 with weighted acquisition. Control spectra was also obtained by irradiating the spins at the appropriate frequency contralateral to the PCr resonance, i.e. at 2.5 ppm. All other acquisition parameters were the same. This CK reaction rate constant was then compared with the standard FID saturation transfer (ST) experiment. ST experiments consisted of 7 spectra with γ-ATP saturated for progressively increasing saturation time. *T*_*sat*_ used in this study were 0, 0.2, 0.4, 0.7, 1.3, 2.5, 4, 6, 9s, d1 = 12s, spectral width = 6 kHz and averages = 8.

Spectra were processed offline using the jMRUI (Java-based magnetic resonance user interface) software. Spectra were fitted in the time domain using a nonlinear least-squares algorithm (AMARES) [38, 39]. Six resonance peaks (Pi: inorganic phosphate; PDE: phosphodiester; PCr: phosphocreatine; and three adenosine triphosphates: α-, β-, γ-ATP) were included in the basis set. The γ-ATP resonance was not included in the basis set for the spectrum where γ-ATP was selectively saturated. *k*_*f*_ was calculate by $T_{1}^{nom}$ approach using Eq 4 and measured *M*_*c*_, *M*_*s*_. *k*_*f*_ was also calculated from fully relaxed ST experiment by fitting PCr resonance intensity to the equation

$$M_{PCr}(t)=\frac{M_{o}}{\tau }(\frac{1}{T_{1}^{int}}+k_{f}e^{-\tau .t})$$ (5)

where $\tau =1/T_{1}^{int}+k_{f}$ is the apparent relaxation rate constant. The CK reaction rate constant was then determined from

$$k_{f}=(\frac{M_{o}-M_{ss}}{M_{o}})/T_{1}^{int}$$ (6)

This equation is mathematically equivalent to Eq 2.

### Studies in the heart

Seven healthy volunteers with no know history of heart disease (6 men and 1 women) age = 24.6 ± 4 years (mean ± SD) and weight = 68.6 ± 16.7 Kg participated in the study. The subjects were placed supine in the magnet and proton localizer images were acquired as previously described. The surface coil was positioned on the chest with the center of the coil just below the mitral valve of the heart. A small fiducial placed at the center of the RF coil was used as a marker to adjust the position of the coil relative to the heart. A non-localized ^31^P spectrum was then acquired and the RF transmit frequency was centered on the PCr resonance.

$T_{1}^{nom}$ experiments used 1D-ISIS/2D-CSI pulse sequence with *T*_*sat*_ = 1.8s, TR = 2.8s, FOV = 16 cm × 16 cm, acquisition matrix 8 × 16 which was interpolated to 16 × 16, slice thickness = 3 cm. spectral width = 6 kHz, 512 spectral points, 16 averages with weighted acquisition. CSI images were reconstructed offline in MATLAB and the spectra from selected voxels was analyzed using AMARES in jMRUI. The spectral model consisted of γ, α, β-ATP peaks, PCr, PDE, and 2,3-diphosphoglycerate [2,3 DPG]. *k*_*f*_ was calculated using Eq 4 and PCr peak area determined from AMARES fitting.

## Results

**Fig 2:**
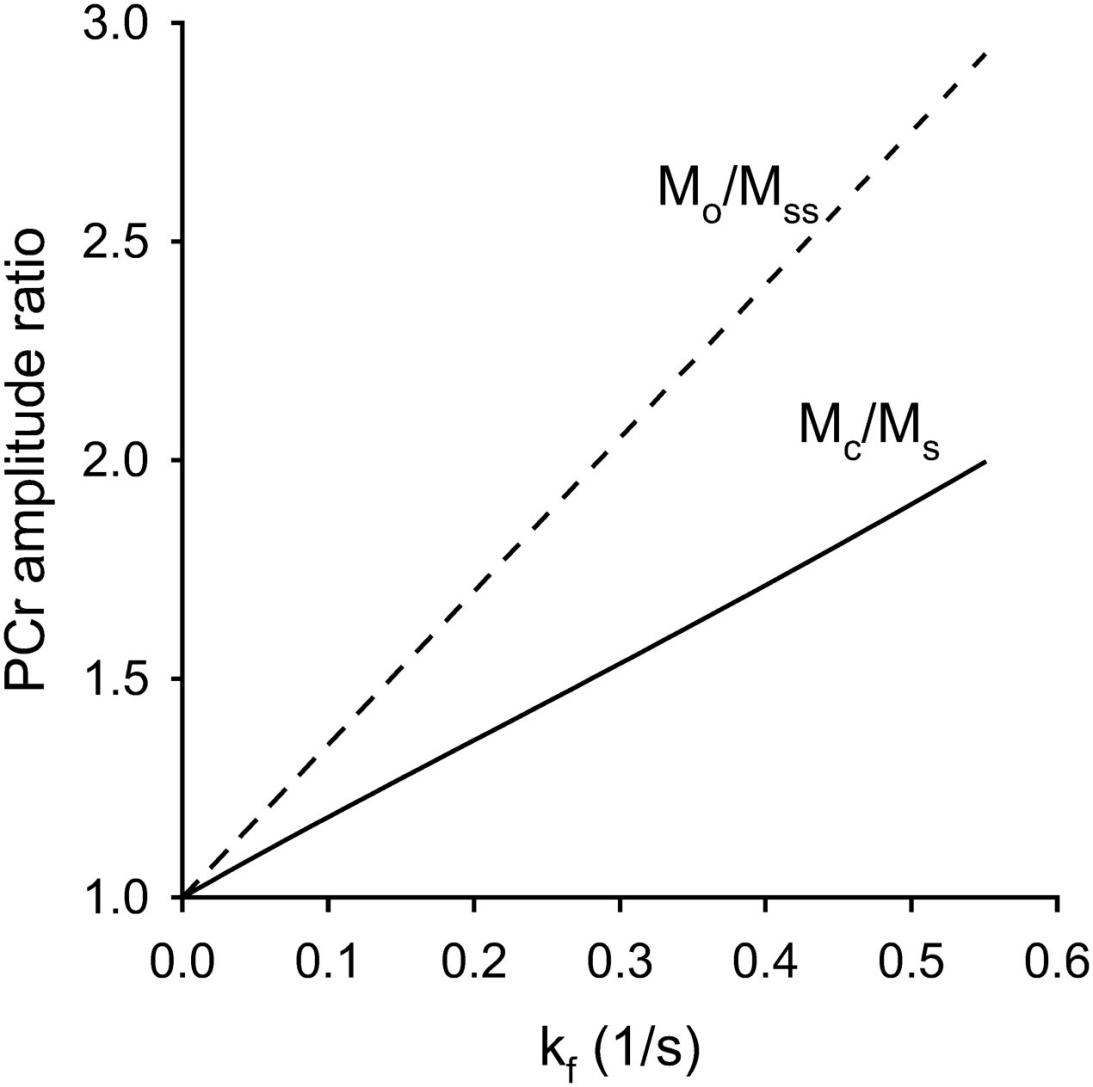
Simulated M_o_/M_ss_ (under fully relaxed conditions) and M_c_/M_s_ ratio vs k_f_ for creatine kinase reaction showing linear relationship and same intercept. The parameters for simulation were: flip angle = 90°, TR = 2.8 s, T_sat_ = 1.8 s, PCr:ATP = 2.0, $T_{1}^{PCr}=4.5\;\text{s}$ and $T_{1}^{ATP}=1.8\;\text{s}$. $T_{1}^{nom}$ determined for these spin parameters is 1.9 s.

Fig 2 shows the numeric simulation obtained with the acquisition parameters used in this study. *M*_*c*_*/M*_*s*_ has shown to have a linear relationship with *k*_*f*._ $T_{1}^{nom}$ determined as slope of the curve is found to be 1.9 sec. Supporting document shows the array of calculated $T_{1}^{nom}$ for a range of spin density parameters (S4 Fig).

**Fig 3:**
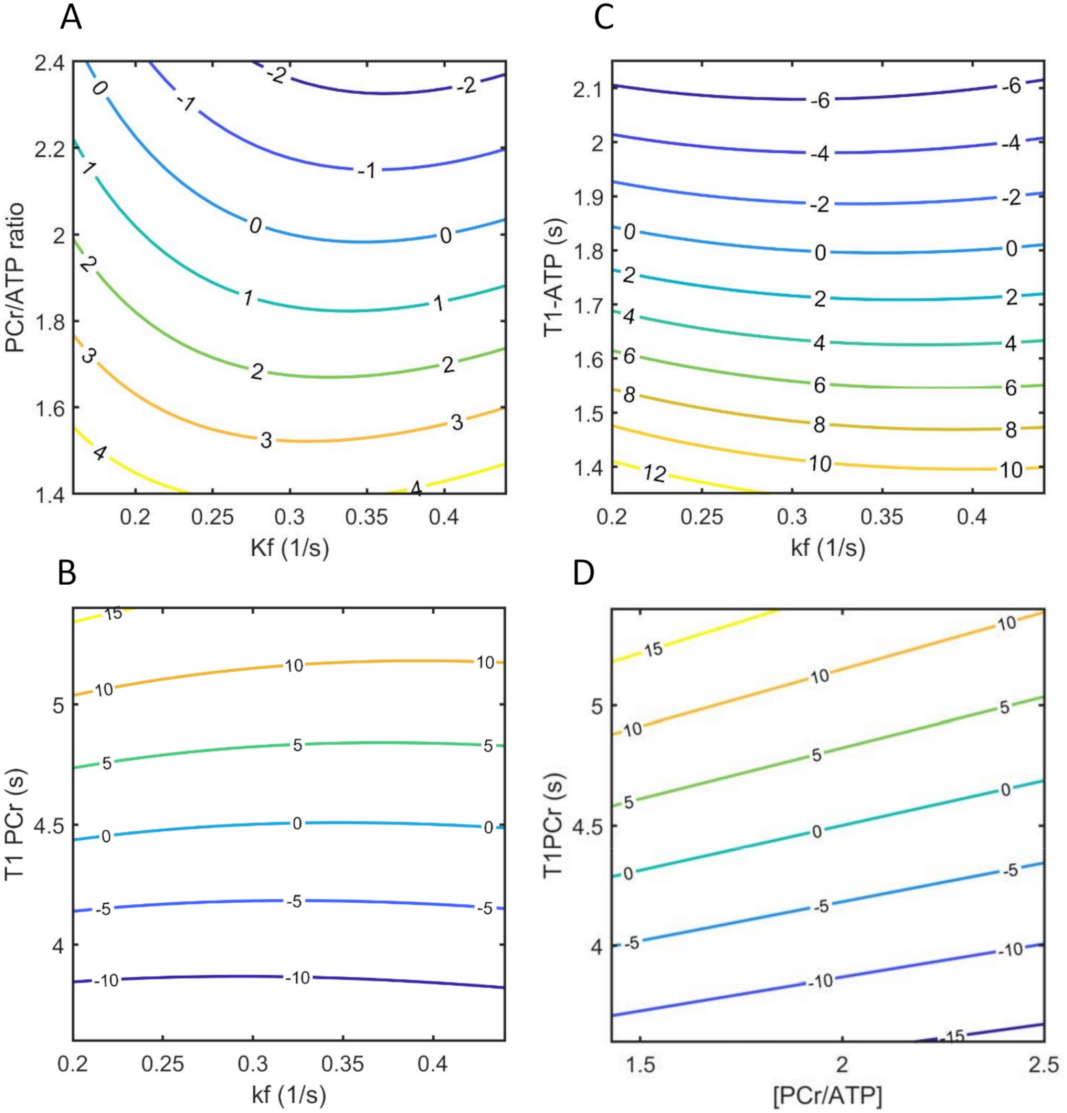
Numeric simulation of percentage error in *k*_*f*_ calculations due to physiological variations in spin parameters. (A) error due to variation in the PCr/ATP ratio (B) error due to changes in $T_{1}^{PCr}$ (C) error due to variations in $T_{1}^{ATP}$ (D) error due to variations in both PCr/ATP ratio and $T_{1}^{PCr}$, *k*_*f*_ was fixed at 0.3 (s^-1^) for this simulation. Parameters used for these numeric simulations were the same as given in Fig 2.

$T_{1}^{nom}$ allows calculation of *k*_*f*_ based on 2 measurements from control (*M*_*c*_) and saturated (*M*_*s*_) spectra. Once $T_{1}^{nom}$ is determined and fixed for given experimental conditions, any physiological and pathological variations in spin parameters will introduce errors in calculated *k*_*f*_. Errors due to variations in spin parameters for a given experimental protocol are shown in Fig 3. The spin parameters were (*PCr*/*ATP*, $T_{1,PCr}^{int}$, and $T_{1,ATP}^{int}$) allowed to vary over a wide range and simulations were used to determine *M*_*c*_ and *M*_*s*_. Calculated *k*_*f*_ and errors were determined based on $T_{1}^{nom}=1.9\;s$ as used in this study. Our simulations show that the relative error in *k*_*f*_ calculation due to unknown pool size ratio and intrinsic relaxation time of metabolites is expected to be less than 10% (Fig 3).

Slice selection is achieved by 1D-ISIS scheme using GOIA-WURST (16, 4) inversion pulse**s** and in plane spatial localization was achieved by 2D-CSI phase encoding (Fig 1). Slice selection tested by 1D-dimensional MR spectrum shows highly selective slice excitation (S1 Fig). The use of broadband GOIA pulse for slice selection enabled highly selective slice shape with negligible contamination from areas outside the slice. The pulse also minimized chemical shift displacement error (CSDE) and corresponds to ±5% between PCr and γ-ATP at 7T. Phantom results showed that OVS is effective in suppressing the tissue signal from near the surface (S2 Fig). This was further tested for in vivo human studies (S3 Fig).

**Fig 4:**
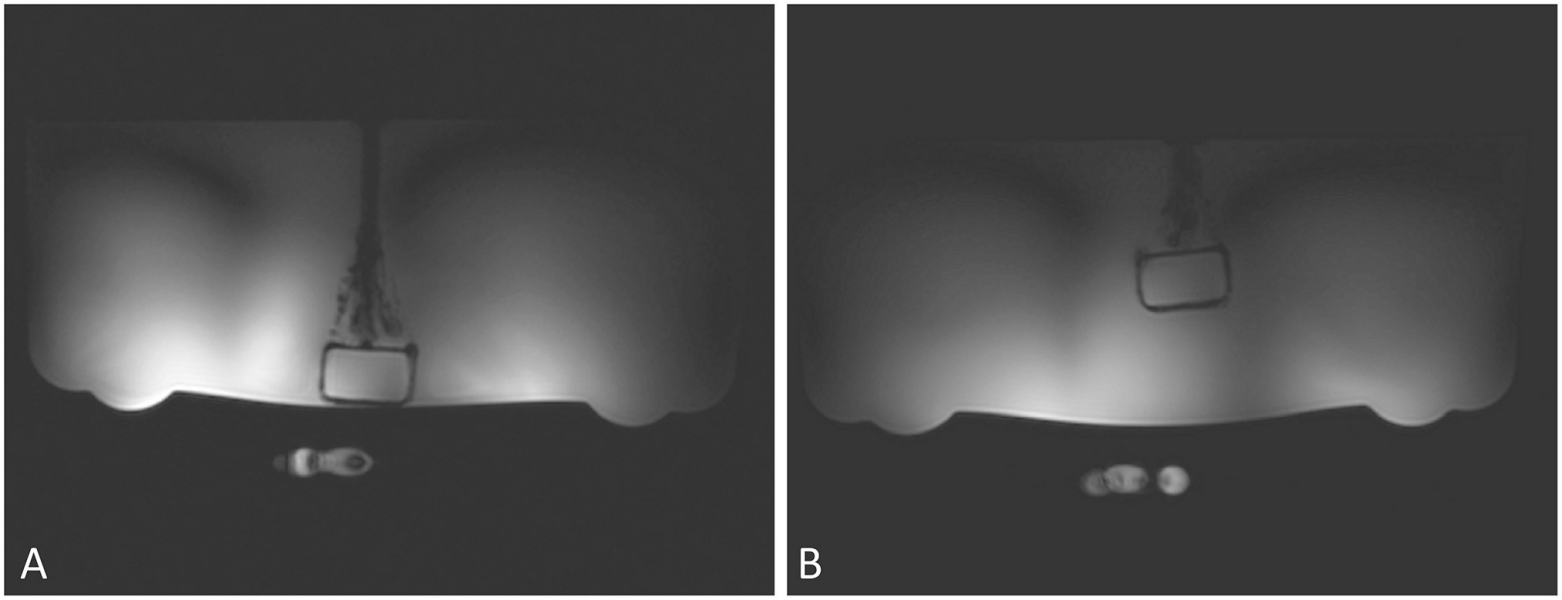
Scout images of the phantom arrangement for T1 measurements. Large beaker is filled with 75 mM sodium chloride and the vial contains 0.5 M solution of sodium phosphate. Fiducial marking the center of the radiofrequency coil is visible in the images. Sodium phosphate vial was held at 3 cm (a) and 7 cm (b) from the surface of RF coil.

Layout of the phantom for T1 measurements is shown in Fig 4. Results from phantom experiments are summarized in Table 1. T1 measured by inversion recovery pulse sequence was 8.87 s. T1 measured ranged from 7.3 s to 10.6 s which would approximately correspond to flip angles ranging from 77° to 106°. T1 measurements on resonance agree with the IR T1 measurements. The errors increased as shown by increased standard deviation. Previous study has shown that this would results in a few percent error in calculating *k*_*f*_ [18].

**Table 1:** Saturation recovery T1 measurement (mean ± standard deviation, 3 measurements each). T1 of phantom was 8.87 s measured with inversion recovery pulse sequence.

Off resonance	Vial Phantom	
	Surface	deep
0 Hz	9.02 ± 0.06	9.17 ± 0.42
-150 Hz	9.97 ± 0.46	10.03 ± 0.51
+150 Hz	8.60 ± 0.70	8.41 ± 1.55

**Fig 5:**
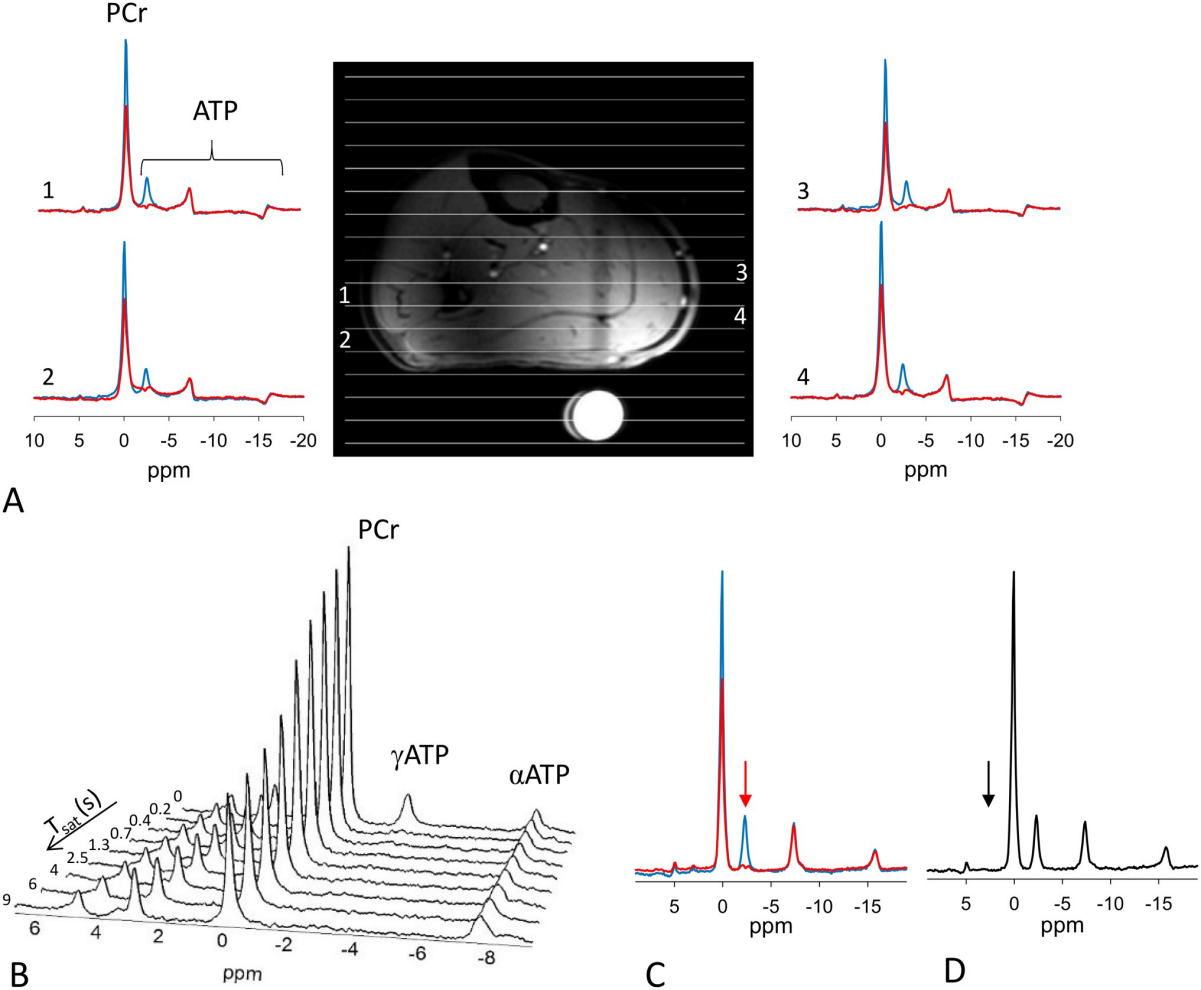
Skeletal muscle *k*_*f*_ determined by conventional saturation transfer approach and $T_{1}^{nom}$ approach with 1D-ISIS/1D-CSI localization. (A) MR image of calf muscle showing the locations of the 1D-CSI voxels. (B) Series of spectra for conventional saturation transfer approach. The peak localization are as α-ATP at -7.6 ppm, γ-ATP at -2.5 ppm, PCr at 0.0 ppm, phosphodiester (PDE) at 3.2 ppm and inorganic phosphates (Pi) at 5.2 ppm. γ-ATP peak is visible in the top spectrum and is selectively saturated for other spectra. Saturation time was progressively increased and PCr peak intensity decreases with the increasing duration of saturation of γ-ATP. (C) Stack of spectra showing M_c_ (without ATP saturation, right) and M_s_ (with ATP saturation, left) using $T_{1}^{nom}$ method.

Validation of the technique was demonstrated in skeletal muscle (Fig 5). Fig 5b shows that in the saturation transfer experiment, the PCr magnetization falls exponentially when γ-ATP was progressively saturated. Eq 6 was used to calculate the CK reaction rate constant. *k*_*f*_ determined from 1D-CSI and $T_{1}^{nom}$ method agrees with the gold standard (Table 2). When control irradiation is applied, no spillover is seen in the PCr resonance intensity (S5 Fig).

**Table 2:** CK reaction rate constants measured in skeletal muscle and myocardium.

	# of subjects	Method	# of voxels	k_f_ (s^-1^)
Leg	3	Saturation Transfer	3	0.31 ± 0.02
		$T_{1}^{nom}$	12	0.30 ± 0.04
Heart	7	$T_{1}^{nom}$	24	0.29 ± 0.05

**Fig 6:**
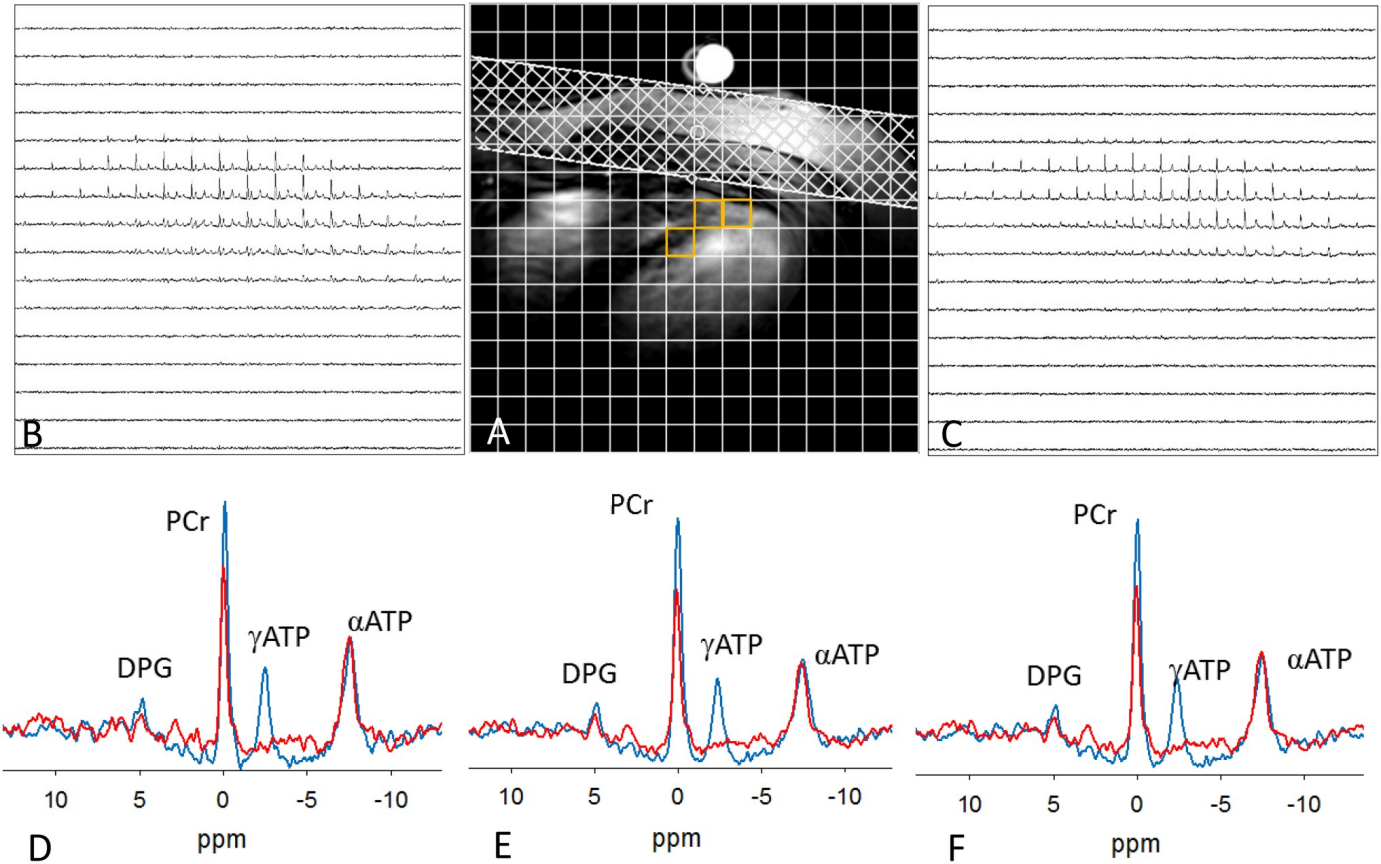
Measurement of in vivo 31P MRS saturation transfer in human heart at 7T. (a) 1H scout image highlighting the typical location of voxel (orange) chosen for analysis. (b) 31P CSI from the slice (b) control and (c) saturation conditions. (d, e, f) Typical 31P magnetization transfer spectra from selected voxels. M_c_ (control) is shown in blue and M_s_ (γ-ATP saturation) is in red. The PCr signal is reduced when γ-ATP is saturated.

Fig 6 demonstrates the 31P spectra obtained from a representative subject. The spectra form the heart was acquired with and without γ-ATP saturation. Anatomic image of heart anatomic image showing CSI grid (Fig 6a). A fiducial marking the center of the RF coil is can be seen in the image. Spectra arising from 3 representative voxels without (M_c_—blue) and with (M_s_—red) γ-ATP saturation shows a reduction in the PCr peak with γ-ATP saturation. Complete saturation of γ-ATP resonance was achieved. Three to four representative voxels were chosen from each subject, and Eq 4 was used to calculate *k*_*f*_. 31P MR spectra from all selected voxels can be found in the supporting information (S6 Fig) yielding 0.29 ± 0.05 s^-1^ (Table 2) which agrees with previously reported values in the myocardium [20, 21, 40–42].

## Discussions

In this work, we demonstrated a fast method for measuring spatially localized CK reaction kinetics in human myocardium at 7T and results in significant reduction in total data acquisition time over conventional saturation transfer approach. The approach was verified in skeletal muscle validating that $T_{1}^{nom}$ method yields the CK reaction rate constant in human leg comparable to the conventional saturation transfer method. The *k*_*f*_ measured in the cardiac experiments are also similar to the published values in human heart. The spatial localization approach used in this study enables precise slice localization with a surface coil as well as reducing the chemical shift displacement errors. The time required for the study, 60 minutes, is less than 90 minutes by 3D CSI BOAST technique previously reported. The time required for actual CK flux data acquisition is 35 minutes and can be further optimized for given application. In summary, the approach enables acquisition of 2D spatially encoded myocardial CK reaction kinetics in clinically acceptable times.

In this technique, we addressed several issues in myocardial ^31^P signal localization using surface coil. Slice selection with conventional amplitude modulated pulses is difficult for surface coil excitations. B_1_ inhomogeneities will affect the slice localization efficiency and lead to both greatly reduced sensitivity with depth and increased outer volume contamination. We employed 1D ISIS technique using GOIA pulses for slice excitation and in-plane spatial localization was achieved by 2D CSI. This approach (GOIA-1D-ISIS/2D-CSI) has been previously demonstrated for brain and liver studies and provides excellent signal localization [24]. The broadband GOIA pulses used in this study significantly reduced chemical shift displacement error for ^31^P MRS at 7T [25]. The excellent inversion profile and slice localization was demonstrated in a phantom study. In comparison to conventional adiabatic inversion pulses, the GOIA pulses of similar bandwidth require significantly lower B_1_. This allows better penetration when using a surface coil and is important for cardiac studies where the heart lies beneath the chest muscles. The duration of the phase-encoding gradients was also minimized which enables a short TE down to 0.6 ms, which minimized the baseline rolling artifacts in the spectrum.

$T_{1}^{nom}$ approach allows for measurement of *k*_*f*_ with arbitrary flip angle and repetition time however the accuracy of *k*_*f*_ calculation based on the $T_{1}^{nom}$ method would be affected by flip angle variation. Arbitrary flip angle for ^31^P in myocardium with surface coils is not possible without prohibitively long B1 mapping protocols. The estimates at best may be erroneous and not feasible for in vivo human studies. Such errors can be minimized by using small-angle adiabatic (BIR4/BIRP) pulses, however BIR4/BIRP pulses are not feasible at 7T due to the limitations of radiofrequency power requirements [43]. Recently the feasibility of AHP excitation for human heart at 7T has been demonstrated which makes calibration easier when surface coils are used [37]. We used AHP pulses as a simple technique for overcoming the need for pulse calibration and once calibrated in pilot experiments no further calibration of the pulses was needed for studies.

One important consideration in cardiac ^31^P MRS is the suppression of unwanted (potentially contaminating) signals from overlying skeletal muscle. Two techniques, crusher coil and B1-insensitive train to obliterate signal (BISTRO), have been previously demonstrated to suppress unwanted signal from the chest muscles in cardiac ^31^P MRS at 7T [44]. The crusher coil approach uses external hardware which is a potential source of noise. BISTRO is very SAR intensive and makes its use prohibitive with saturation transfer studies. In the only other published study of CK flux measurements at 7T, there was no suppression of signal from the chest muscles, and localization only relied on phase encoded CSI. This can potentially lead to contaminating signal from surface tissue in the sensitive region of the coil. In order to reduce signal contamination, we used standard outer volume saturation (OVS) technique using vendor optimized amplitude modulated sinc pulses designed for outer volume suppression. The performance of the signal suppression was calibrated on phantom and it gave 40% residual signal in the first 40 mm of the surface tissue. This suppression is on par with the crusher coil (34% residual signal) and BISTRO (50% residual signal) approaches presented earlier [44]. This method is simple to use and SAR efficient alternative to BISTRO without using any external hardware.

Errors in quantification of reaction rate constant may arise from direct spillover irradiation of PCr and incomplete saturation of γ-ATP. Increased chemical shift dispersion at 7T improves the ability to saturate γ-ATP, whilst minimizing the spillover to the PCr resonance. Negligible spillover irradiation of PCr was demonstrated (S6 Fig). The saturation pulse was calibrated to saturate γ-ATP at a distance of ~ 8cm from the coil. There was no significant residual γ-ATP observed in the in vivo heart spectra. Hence, no correction was needed for these calculations.

The major obstacle to spatially localized ^31^P MRS of CK reaction rate calculation is the low signal-to-noise ratio (SNR) because of the intrinsically low nuclear gyromagnetic ratio of ^31^P and the low concentration of phosphorus metabolites. $T_{1}^{nom}$ method relies on linear relationship between *M*_*c*_/*M*_*s*_ and *k*_*f*_, and the simulations have shown that this linear relationship is maintained for range of acquisition parameters [18]. This $T_{1}^{nom}$ approach can be considered as improved version of conventional saturation transfer approach allowing for the optimization of the protocol for best SNR per unit time.

A limitation of $T_{1}^{nom}$ method is that calculation of *k*_*f*_ depends on knowledge of the ratio of metabolite pool sizes (PCr/ATP ratio) and intrinsic *T*_1_ of metabolites. The extensive previous studies on CK kinetics have suggested that the intrinsic *T*_1_ is constant among subjects and across species regardless of physiological and pathological conditions. Intrinsic *T*_1_ was also shown to be independent of reaction rate change throughout CK inhibition process in myocardium. The PCr/ATP ratio is known to change (decrease) with heart dysfunction or disease. For the given acquisition parameters, we have shown that a broad range of variations in spin parameters would result in less than 10% of *k*_*f*_ measurement error using $T_{1}^{nom}$ method. Previous studies have shown that *k*_*f*_ ranges from ~ 0.33 (1/s) in normal human myocardium to 0.17 (1/s) in heart failure [10, 45]. The simulation were done to cover this spread in *k*_*f*_. Results also shows that when PCr/ATP is decreased due to pathology the $T_{1}^{nom}$ approach will overestimate *k*_*f*_. In the limit where PCr/ATP is 1.4 and *k*_*f*_ = 0.2 (1/s), $T_{1}^{nom}$ will predict *k*_*f*_ = 0.209 (1/s) which is expected to be below the experimental errors and physiological differences (Fig 3). In case the spin parameters are not known or expected to vary, this approach can be combined with global measurements of spin parameters. For example, global measurement of PCr/ATP ratio and intrinsic *T*_*1*_ can be measured quickly and $T_{1}^{nom}$ can be determined iteratively to further reduce errors measuring CK reaction rate constant.

A limitation of this study is that metabolite pool size ratios were note determined. Recently it was demonstrated that $T_{1}^{nom}$ can be extended to simultaneously quantify reaction constants as well as metabolite pool size ratios [46]. This requires application of known flip angles, which is technically challenging at 7T with surface coil. Clarke et al. have demonstrated an elegant technique to map flip angles using Bloch-Siegert phase shift produced by off-resonance RF pulse [22]. Future studies should explore this or other flip angle mapping techniques to determine all components of CK reaction simultaneously. Our simulation results show that the errors due to physiological variations in global pool size ratios are expected to be below experimental errors. These errors can be further minimized by measuring the global pool size ratio measurements which is a limitation of this study. In healthy individuals (subjects for this study) the pool size ratios are not expected to vary significantly. However if patient population is to be examined it is highly recommended to measure the pool size ratios and incorporate in the calculations.

RF power limitations at 7T can cause flip-angle dependent errors in magnetization transfer experiments. T1 measurements on phantom placed along the axis of in the up to a depth of ~8 cm shows agreement with the inversion recovery to within ~3% for spins on resonance but increased to ~13% for spins 150 Hz off resonance. This can lead to errors in calculating CK reaction kinetics. Although not exclusively done in this study, previous simulations have shown that this would result in few percent error in estimating forward CK reaction rate constant. We examined the voxels in the anterior and septal wall of the myocardium close to the center axis of the coil (Fig 6) and the measured values are consistent with the previously reported *k*_*f*_ values [20, 21, 41, 45, 47]. This setup is useful for studying anterior and septal wall infarcts. If ones wants to examine voxels distal from the coil axis the adiabatic assumption for RF pulse might not be valid. In this case one has to move the coil center to be positioned over the region of interest or measure the flip angles and apply necessary corrections.

Due to experimental limitations cardiac and respiratory gating is not used with saturation transfer studies of CK flux in heart. Most in vivo human cardiac CK flux studies have been conducted without cardiac and respiratory gating. Respiratory gating can be partially mitigated with subject in prone position which can cause discomfort for participants. Cardiac triggering can be employed however, it will require estimating average saturation time form physiological data. Physiological data is not reliable at 7T due magnetohydrodynamic effects and lead to sources of error in calculating CK flux [10, 15–17, 20, 21]. Several steps were taken to mitigate the effect of motion. The subjects were constantly instructed for shallow breathing. All scouts were acquired with free-breathing to estimate the extent of motion and slice locations were chosen to account for the motion. Even with these precautions respiratory and cardiac motion can lead to voxel blurring and increased spectral linewidths and is unavoidable and is a limitation that needs to be addressed in future studies.

The studies in human skeletal muscle demonstrated that the spatially localized $T_{1}^{nom}$ method yields the same values of *k*_*f*_ as the unlocalized conventional saturation transfer method. Localized conventional saturation transfer studies are too time consuming for human studies, thus the $T_{1}^{nom}$ approach allows measurement of localized CK reaction kinetics in clinically relevant acquisition times. The values of *k*_*f*_ = 0.31 ± 0.02 (s^-1^) by saturation transfer and *k*_*f*_ = 0.30 ± 0.04 (s^-1^) by $T_{1}^{nom}$ method were similar to the values reported previously [15, 20, 21, 48, 49]. Although 1D CSI was demonstrated in the skeletal muscle, the technique was easily adapted to 2D chemical shift imaging studies in the heart. The *k*_*f*_ measured in cardiac studies closely matched the literature values (Table 2). The simulation studies show that the error in the measured *k*_*f*_ is expected to be small and was confirmed by the experiments in the leg. The total date using $T_{1}^{nom}$ method is 35 minutes in contrast a similar conventional saturation transfer experiment using a conventional saturation transfer experiment will take approximately 3 hours, approximately 83% saving in time.

This approach is advantageous over other rapid saturation transfer methods which use multiple flip angles for calculating the *k*_*f*_ and thus more vulnerable to flip angle variation. Another advantage of this strategy is that the sequence can be optimized to reduce the saturation time for *M*_*s*_ measurement. Shorter saturation time is beneficial to reduce SAR at high field when human subjects are involved. The other advantage is that shorter saturation time reduces spillover of the saturation pulse on PCr thus improving accuracy. The performance of the method can be further optimized for given application and the optimization strategies to improve performance have been previously described in detail [18].

In summary, we have demonstrated a strategy to reduce acquisition time in saturation transfer chemical shift imaging studies of CK reaction kinetics. The approach allows measurement for rapid acquisition of ^31^P saturation transfer MRS under partially relaxed conditions and enables fast mapping of *k*_*f*_ in myocardium. We also implemented the technique with GOIA-1D-ISIS/2D-CSI signal localization enabling accurate slice localization suitable for surface coil and high field studies. This work enables broad applications for in vivo studies of energetics and mitochondrial function in heart and other organs in clinically relevant acquisition time.

## Supporting information

S1 Fig Performance of 1D-ISIS slice excitation performance was determined in cylindrical (diameter = 16 cm and length = 26 cm) phantom containing 100 mM sodium phosphate. A one-dimensional profile was acquired in a plane parallel to the surface of the coil. (A) High resolution image of the phantom and slice location. The ^31^P/1H RF coil was placed under the phantom. (B) Experimentally measured ^31^P excitation slice profile shown in red demonstrates excellent localization with minimal side bands. Non-localized profile (GOIA pulses turned off) is shown in black. The signal decays towards the edges is due to loss of sensitivity of the surface coil.
(DOCX)

S2 Fig Performance of outer volume saturation pulse. A one-dimensional profile was acquired in a plane perpendicular to the surface of the coil. Localizer image has been rotated 90 degrees for display purposes. Experiment details are provided in the manuscript. Blue graph shows the profile of the excited slice measured perpendicular to the plane of the RF coil in absence of OVS slab. The red profile shows the signal profile in presence of the OVS slab as a function of flip angle. Flip angle from 20° to 60° is efficient in suppressing signal form the phantom. Flip angle of 45° was chosen for all in vivo experiments.
(DOCX)

S3 Fig (a) Reference image of the heart and placement of OVS. Fiducial marking the center of the radiofrequency coil is visible in the images. (b) Spectrum without OVS shows a large PCr peak relative to ATP (PCr:ATP > 3:1) indicating signal arising from the chest muscles. (b) With OVS the PCr peak height is significantly reduced suggesting suppression of signal from the chest tissue.
(DOCX)

S4 Fig$T_{1}^{nom}$ calculations for various spin density parameters.Experimental parameters were kept constant with TR = 2.8 s and *T*_*sat*_ = 1.8 s and 90° flip angle. $T_{1}^{nom}$ was determined by numerically calculating *M*_*c*_ and *M*_*s*_ according to Eq 1 and linear regression was used to calculate $T_{1}^{nom}$.(DOCX)

S5 Fig (A) Representative spectra for skeletal muscle from saturation transfer experiment. (B) Control spectrum with selective RF irradiation at 2.5 ppm. The arrows identify the frequency of the saturating irradiation. (C) Representative spectra when the γ-ATP peak is saturated.
(DOCX)

S6 Fig MR spectra from all selected voxels used in analysis. (DOCX)
