# Supplementary material for: Creatine kinase rate constant in the human heart at 7T with 1D-ISIS/2D CSI localization
Source: PLoS One. 2020 Mar 19;15(3):e0229933. doi: 10.1371/journal.pone.0229933 (PMC7081998; doi:10.1371/journal.pone.0229933)
Supplement: S1 Fig — A one-dimensional profile was acquired in a plane parallel to the surface of the coil. (A) High resolution image of the phantom and slice location. The 31P/1H RF coil was placed under the phantom. (B) Experimentally measured 31P excitation slice profile shown in red demonstrates excellent localization with minimal side bands. Non-localized profile (GOIA pulses turned off) is shown in black. The signal decays towards the edges is due to loss of sensitivity of the surface coil. (DOCX) [file pone.0229933.s001.docx]

**
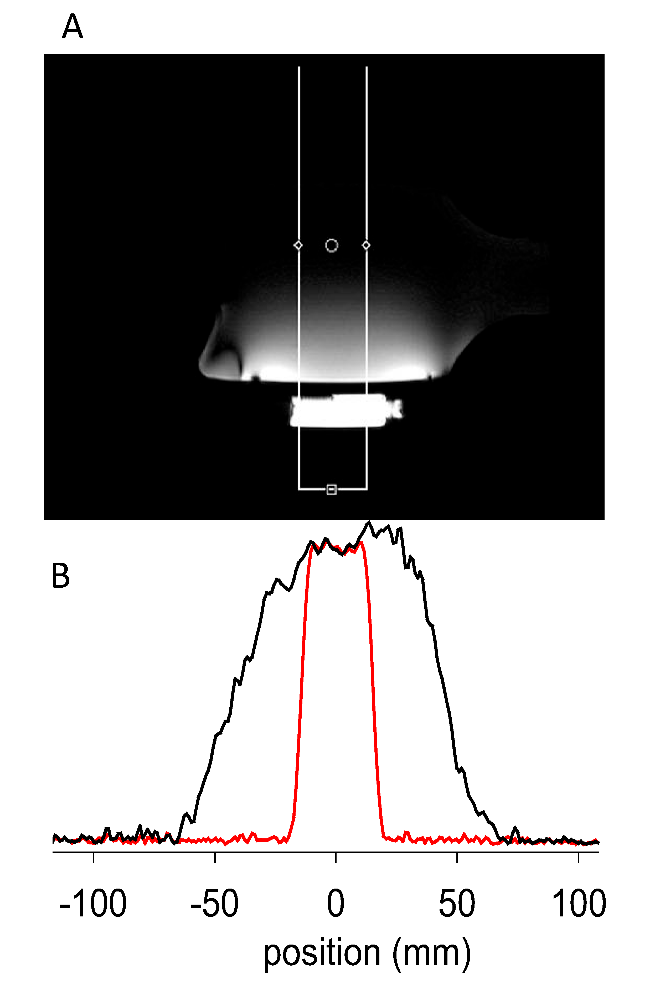
**

**S1 Figure 1:** Performance of 1D-ISIS slice excitation performance was determined in cylindrical (diameter =16 cm and length = 26 cm) phantom containing 100 mM sodium phosphate. A one-dimensional profile was acquired in a plane parallel to the surface of the coil. (A) High resolution image of the phantom and slice location. The ^31^P/1H RF coil was placed under the phantom. (B) Experimentally measured ^31^P excitation slice profile shown in red demonstrates excellent localization with minimal side bands. Non-localized profile (GOIA pulses turned off) is shown in black. The signal decays towards the edges is due to loss of sensitivity of the surface coil.
