# Supplementary material for: Creatine kinase rate constant in the human heart at 7T with 1D-ISIS/2D CSI localization
Source: PLoS One. 2020 Mar 19;15(3):e0229933. doi: 10.1371/journal.pone.0229933 (PMC7081998; doi:10.1371/journal.pone.0229933)
Supplement: S2 Fig — A one-dimensional profile was acquired in a plane perpendicular to the surface of the coil. Localizer image has been rotated 90 degrees for display purposes. Experiment details are provided in the manuscript. Blue graph shows the profile of the excited slice measured perpendicular to the plane of the RF coil in absence of OVS slab. The red profile shows the signal profile in presence of the OVS slab as a function of flip angle. Flip angle from 20° to 60° is efficient in suppressing signal form the phantom. Flip angle of 45° was chosen for all in vivo experiments. (DOCX) [file pone.0229933.s002.docx]

**S1 Figure 2:** Performance of outer volume saturation pulse. A one-dimensional profile was acquired in a plane perpendicular to the surface of the coil. Localizer image has been rotated 90 degrees for display purposes. Experiment details are provided in the manuscript. Blue graph shows the profile of the excited slice measured perpendicular to the plane of the RF coil in absence of OVS slab. The red profile shows the signal profile in presence of the OVS slab as a function of flip angle. Flip angle from 20° to 60° is efficient in suppressing signal form the phantom. Flip angle of 45° was chosen for all in vivo experiments.
