# Supplementary material for: Creatine kinase rate constant in the human heart at 7T with 1D-ISIS/2D CSI localization
Source: PLoS One. 2020 Mar 19;15(3):e0229933. doi: 10.1371/journal.pone.0229933 (PMC7081998; doi:10.1371/journal.pone.0229933)
Supplement: S3 Fig — (a) Reference image of the heart and placement of OVS. Fiducial marking the center of the radiofrequency coil is visible in the images. (b) Spectrum without OVS shows a large PCr peak relative to ATP (PCr:ATP > 3:1) indicating signal arising from the chest muscles. (b) With OVS the PCr peak height is significantly reduced suggesting suppression of signal from the chest tissue. (DOCX) [file pone.0229933.s003.docx]

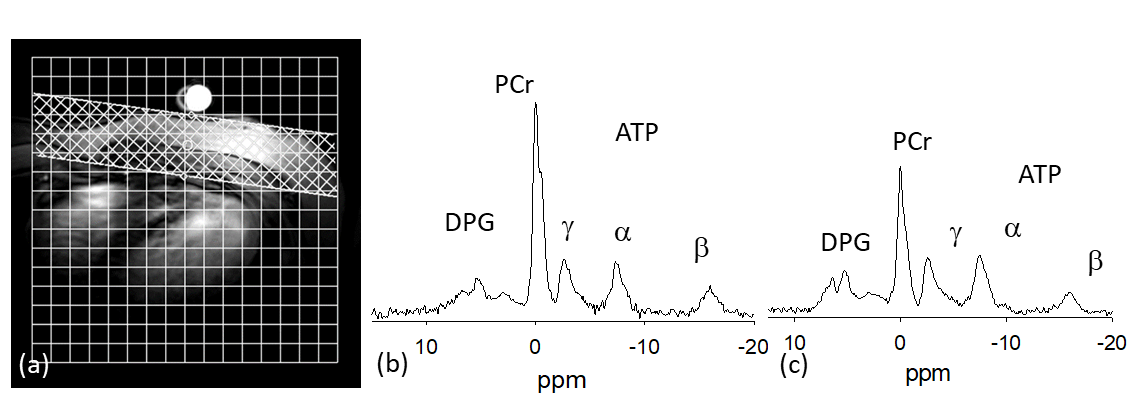


**S1 Figure 3:** (a) Reference image of the heart and placement of OVS. Fiducial marking the center of the radiofrequency coil is visible in the images. (b) Spectrum without OVS shows a large PCr peak relative to ATP (PCr:ATP > 3:1) indicating signal arising from the chest muscles. (b) With OVS the PCr peak height is significantly reduced suggesting suppression of signal from the chest tissue.
