# Supplementary material for: Creatine kinase rate constant in the human heart at 7T with 1D-ISIS/2D CSI localization
Source: PLoS One. 2020 Mar 19;15(3):e0229933. doi: 10.1371/journal.pone.0229933 (PMC7081998; doi:10.1371/journal.pone.0229933)
Supplement: S4 Fig — Experimental parameters were kept constant with TR = 2.8 s and Tsat = 1.8 s and 90° flip angle. T1nom was determined by numerically calculating Mc and Ms according to Eq 1 and linear regression was used to calculate T1nom. (DOCX) [file pone.0229933.s004.docx]

**S1 Figure 4:** $T_{1}^{nom}$ calculations for various spin density parameters. Experimental parameters were kept constant with TR = 2.8 s and $T_{sat}=1.8$ s and 90° flip angle. $T_{1}^{nom}$ was determined by numerically calculating *M_c_* and *M_s_* according to equation 1 and linear regression was used to calculate $T_{1}^{nom}$
