# Supplementary material for: Creatine kinase rate constant in the human heart at 7T with 1D-ISIS/2D CSI localization
Source: PLoS One. 2020 Mar 19;15(3):e0229933. doi: 10.1371/journal.pone.0229933 (PMC7081998; doi:10.1371/journal.pone.0229933)
Supplement: S5 Fig — (A) Representative spectra for skeletal muscle from saturation transfer experiment. (B) Control spectrum with selective RF irradiation at 2.5 ppm. The arrows identify the frequency of the saturating irradiation. (C) Representative spectra when the γ-ATP peak is saturated. (DOCX) [file pone.0229933.s005.docx]

**S1 Figure 5:** (A) Representative spectra for skeletal muscle from saturation transfer experiment. (B) Control spectrum with selective RF irradiation at 2.5 ppm. The arrows identify the frequency of the saturating irradiation. (C) Representative spectra when the γ-ATP peak is saturated.
