# Supplementary figures and images for: Creatine kinase rate constant in the human heart at 7T with 1D-ISIS/2D CSI localization
Source: PLoS One. 2020 Mar 19;15(3):e0229933. doi: 10.1371/journal.pone.0229933 (PMC7081998; doi:10.1371/journal.pone.0229933)

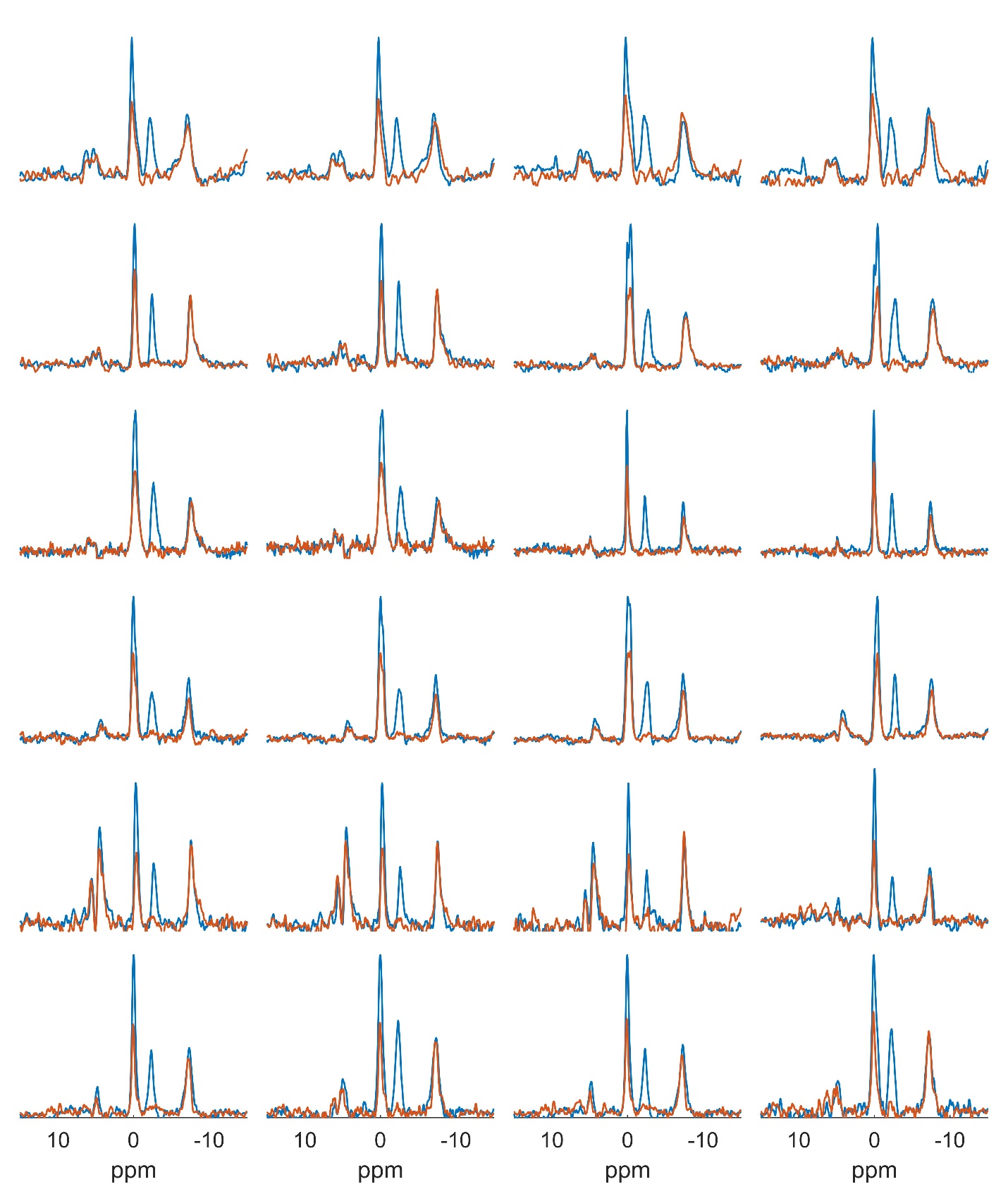


**S1 Figure 6:** MR spectra from all selected voxels used in analysis.

Supplement: S6 Fig — (DOCX) [file pone.0229933.s006.docx]
